# Supplementary material for: Targeting FAK/PYK2 with SJP1602 for Anti-Tumor Activity in Triple-Negative Breast Cancer
Source: Curr Issues Mol Biol. 2023 Aug 24;45(9):7058–74. doi: 10.3390/cimb45090446 (PMC10528299; doi:10.3390/cimb45090446)
Supplement: Supplementary file 1 [file cimb-45-00446-s001.zip › cimb-2562990-SI.pdf]

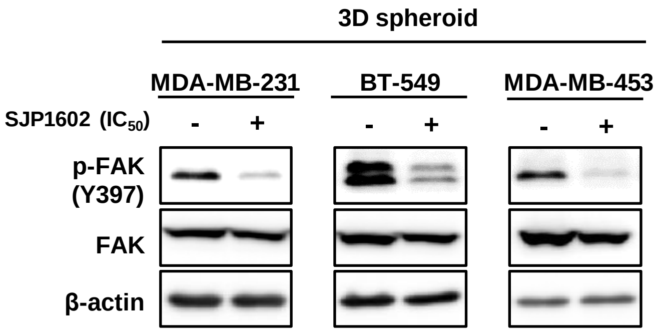

Supplementary Figure S1. Effect of SJP1602 on TNBC Cells. TNBC cells were treated with SJP1602 at concentrations equivalent to the IC_{50} for pFAK. Western blot analysis was utilized to assess the expression and phosphorylation of FAK proteins.

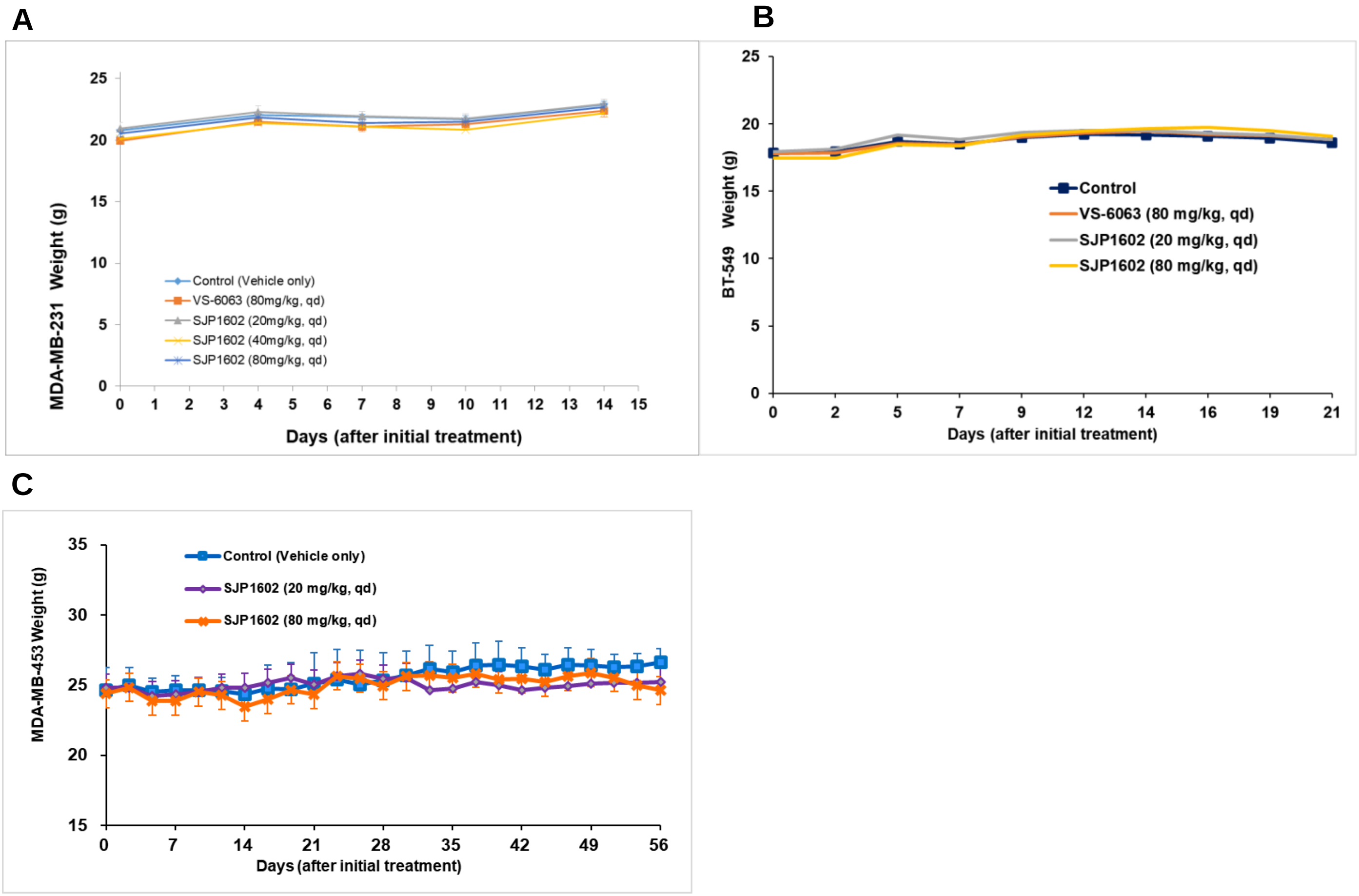

Supplementary Figure S2. Changes in Body Weight of TNBC Xenograft Mouse Models Following Treatment with SJP1602. Changes in mouse body weights during experiment. (A) MDA-MB-231, (B) BT-549, and (C) MDA-MB-453.

	VS-6063 @ 1 µM	GSK-2256098 @ 1 µM	SJP1602 @ 1 µM
FAK(h)	-6	-1	3
Pyk2(h)	6	60	4
Met(h)	42	76	21
ALK(h)	96	73	28
Aurora-A(h)	12	43	48
CHK2(h)	71	59	51
Fes(h)	7	49	54
Mer(h)	7	97	60
IR(h), activated	30	76	61
CHK1(h)	58	85	62
Lck(h)	30	110	63
Fer(h)	31	105	68
MELK(h)	9	91	74
Ron(h)	72	91	75
Abl(h)	57	99	76
Flt3(h)	6	108	77
CSK(h)	88	92	83
Bmx(h)	47	105	84
CDK5/p35(h)	10	94	84
Yes(h)	56	99	84
Blk(h)	62	117	85
CDK2/cyclinA(h)	28	94	85
Fms(h)	67	88	85
PAK4(h)	42	93	85
PI3 Kinase (p120g)(h)	104	101	85
FGFR3(h)	44	91	86
Flt3(D835Y)(h)	7	76	87
Pim-1(h)	86	87	87
CDK1/cyclinB(h)	14	97	88
CK1γ3(h)	84	95	88
EGFR(T790M,L858R)(h)	65	88	88
ARK5(h)	21	92	90
c-RAF(h)	80	72	90
FGFR2(h)	46	98	90
PI3 Kinase (p110d/p85a)(h)	88	115	91
B-Raf(h)	83	99	93
EphB4(h)	67	94	93
PKCι(h)	100	106	93
PKCθ(h)	63	96	93
MAPK2(h)	87	94	95
MST3(h)	33	102	95
NIM1(h)	78	85	95
PKCη(h)	104	97	95
LKB1(h)	89	98	96
cKit(h)	80	93	97
DYRK2(h)	80	52	97
PDGFRα(h)	79	101	97
ATR/ATRIP(h)	92	101	97
PI3 Kinase (p110a(H1047R)/p85a)(h)	101	97	97
DCAMKL3(h)	42	98	98
MEK1(h)	93	101	98
ATM(h)	63	94	98
DNA-PK(h)	85	98	98
PI3 Kinase (p110a(E545K)/p85a)(h)	100	93	98

	VS-6063 @ 1 µM	GSK-2256098 @ 1 µM	SJP1602 @ 1 µM
cSRC(h)	72	88	99
ErbB4(h)	104	81	99
HIPK2(h)	16	94	100
HIPK3(h)	54	106	100
PKBα(h)	101	105	100
PKCα(h)	92	95	100
ZIPK(h)	97	93	100
PI3 Kinase (p110b/p85a)(h)	103	105	100
CK1γ2(h)	64	97	101
KDR(h)	28	88	101
ROCK-I(h)	103	107	101
Wee1(h)	72	88	101
PI3 Kinase (p110a(E542K)/p85a)(h)	104	97	101
DDR2(h)	94	138	102
FGFR4(h)	104	104	102
PDK1(h)	66	117	102
EphA7(h)	41	99	103
IKKε(h)	88	102	103
IR(h)	62	105	103
PKCε(h)	98	112	103
Flt4(h)	-3	67	104
Ros(h)	106	103	104
PI3 Kinase (p110a/p85a)(h)	103	83	104
Axl(h)	57	84	105
B-Raf(V599E)(h)	89	67	105
CK1γ1(h)	81	99	105
EphA2(h)	91	92	105
Hck(h)	99	100	105
IGF-1R(h), activated	74	108	105
Snk(h)	64	113	105
Tie2 (h)	82	108	105
Abl(T315I)(h)	44	92	106
Flt1(h)	21	94	106
Lck(h) activated	58	90	107
DAPK1(h)	94	83	108
BRK(h)	91	67	109
TrkA(h)	2	103	109
FGFR1(h)	43	109	111
Ret(h)	39	117	111
EGFR(h)	105	98	112
ErbB2(h)	122	94	113
PKCµ(h)	100	109	113
TAK1(h)	71	95	113
IGF-1R(h)	55	115	116
PKD2(h)	116	90	116
EGFR(T790M)(h)	82	106	119
PAK1(h)	79	91	119
EGFR(L861Q)(h)	128	69	120
TrkC(h)	13	104	120
cKit(D816H)(h)	94	98	124
p70S6K(h)	98	69	124
ZAP-70(h)	139	105	124
IKKα(h)	125	93	130
EGFR(L858R)(h)	134	33	133
cKit(V560G)(h)	74	104	136
PDGFRα(D842V)(h)	87	119	141

Supplementary Table S1. Kinase Assays for SJP1602. The data show the residual kinase activity after treatment with 1 µM of SJP1602.

Compound	Cytotoxicity on Normal Cell Lines (IC50, nM)				
	CCD-18Co	WI38	Fa2N4	FDF	HEK293T
**SJP1602**	>10,000	>10,000	7,276	9,616	>10,000

Supplementary Table S2. Cytotoxicity Evaluation of SJP1602 on Normal Cell Lines.
